# Bulkley’s Management of Eczema

**Published:** 1875-09

**Authors:** 


The Management of Eczema. By L. Duncan Bulkley, A.M., M.D., New York: G. P. Putnam's Sons. 1875. Octavo; boards; pp. 22.

We doubt not there are many of our readers who may gather new and most useful thoughts, of practical value in the "management" of obstinate and most vexing cases of eczema, from this little brochure of Dr. Bulkley. We extract from the preface a paragraph which will give an insight into his ideas:

"The aim of the following pages is to impress the fact that while eczema is a disease *sui generis*, it is not to be treated abstractly as such, but that rather the patient is to be restored to health; that eczema depends upon many different conditions, and is consequently amenable to the most varied measures, so that its treatment is better styled the *management* of eczema."

